# Supplementary material for: Polyketide synthase gene clusters in the ericoid mycorrhizal fungus Oidiodendron maius: genome-wide prediction, expression profiling, and functional characterization of knockout mutants
Source: Front Plant Sci. 2026 Sep 16;17:1924430. doi: 10.3389/fpls.2026.1924430 (PMC13623903; doi:10.3389/fpls.2026.1924430)
Supplement: Supplementary file 1 [file SupplementaryFile1.zip › Supplementary Materials/Supplementary Folder 1.zip!/Oidma1_AssemblyScaffolds_Repeatmasked/knownclusterblast/cluster54/jgi.p_Oidma1_184949_mibig_hits.html]

| MIBiG Protein | Description | MIBiG Cluster | MiBiG Product | % ID | % Coverage | BLAST Score | E-value |
| --- | --- | --- | --- | --- | --- | --- | --- |
| CCA54216.1 | Aldo-keto\_reductase | BGC0000893 | Chloramphenicol\_biosynthetic\_gene\_cluster | 45 | 62.0553359684 | 116 | 6e-26 |
| EFG07997.1 | Aldo/keto\_reductase | BGC0000843 | Clavams\_biosynthetic\_gene\_cluster | 41 | 70.7509881423 | 110 | 2.5e-24 |
| ADE34525.1 | SsfF | BGC0000269 | SF2575\_biosynthetic\_gene\_cluster | 43 | 62.8458498024 | 109 | 7.4e-24 |
| AFH74311.1 | Cvm1-like\_aldo/keto\_reductase | BGC0001151 | Clavams\_biosynthetic\_gene\_cluster | 42 | 60.8695652174 | 104 | 1.8e-22 |
